# New Books

**Published:** 2008-07

**A Community Guide to Environmental Health**

Jeff Conant, Pam Fadem

Berkeley:Hesperian, 2008. 617 pp. ISBN: 978-0-942364-56-9, $28

**Acid Toxicity and Aquatic Animals**

R. Morris, E.W. Taylor, D.J.A. Brown, J.A. Brown, eds.

New York:Cambridge University Press, 2008. 294 pp. ISBN: 978-0-521-05762-2, $48

**Bending Science: How Special Interests Corrupt Public Health Research**

Thomas O. McGarity, Wendy E. Wagner

Cambridge, MA:Harvard University Press, 2008. 384 pp. ISBN: 978-0-674-02815-9, $45

**Climate Extremes and Society**

Henry F. Diaz, ed.

New York:Cambridge University Press, 2008. 356 pp. ISBN: 978-0-521-87028-3, $140

**Cultivating Science, Harvesting Power: Science and Industrial Agriculture in California**

Christopher R. Henke

Cambridge, MA:MIT Press, 2008. 256 pp. ISBN: 978-0-262-08373-7, $32

**Evaluation of Certain Food Additives and Contaminants**

Sixty-eight Report of the Joint FAO/WHO Expert Committee on Food Additives

Geneva:WHO Press, 2008. 236 pp. ISBN: 978-924-120947-2, $40

**Facing Climate Change Together**

Catherine Gautier, Jean-Louis Fellous, eds.

New York:Cambridge University Press, 2008. 280 pp. ISBN: 978-0-521-89682-5, $80

**From the Corn Belt to the Gulf: Societal and Environmental Implications of Alternative Agricultural Futures**

Joan Iverson Nassauer, Mary V. Santelmann, Donald Scavia, eds.

Washington, DC:RFF Press, 2007. 260 pp. ISBN: 978-1-933115-47-4, $85

**Global Warming: Implications for Freshwater and Marine Fish**

C.M. Wood, D.G. McDonald, eds.

New York:Cambridge University Press, 2008. 441 pp. ISBN: 978-0-521-05789-9, $70

**Life as It Is: Biology for the Public Sphere**

William F. Loomis

Berkeley:University of California Press, 2008. 272 pp. ISBN: 978-0-520-25357-5, $24.95

**Monitoring Ecological Impacts: Concepts and Practice in Flowing Waters**

Barbara J. Downes, Leon A. Barmuta, Peter G. Fairweather, Daniel P. Faith, Michael J Keough, P.S. Lake, Bruce D. Mapstone, Gerry P. Quinn

New York:Cambridge University Press, 2008. 450 pp. ISBN: 978-0-521-06529-0, $70

**New York for Sale: Community Planning Confronts Global Real Estate**

Tom Angotti

Cambridge, MA:MIT Press, 2008. 304 pp. ISBN: 978-0-262-01247-8, $24.95

**Political Theory and Global Climate Change**

Steve Vanderheiden, ed.

Cambridge, MA:MIT Press, 2008. 280 pp. ISBN: 978-0-262-22084-2, $60

**Solar Revolution**

Travis Bradford

Cambridge, MA:MIT Press, 2008. 254 pp. ISBN: 978-0-262-52494-0, $14.95

**Strategic Bargaining and Cooperation in Greenhouse Gas Mitigations**

Zili Yang

Cambridge, MA:MIT Press, 2008. 216 pp. ISBN: 978-0-262-24054-3, $40

**The Atlas of Climate Change: Mapping the World’s Greatest Challenge**

Kirstin Dow, Thomas E. Downing

Berkeley:University of California Press, 2007. 128 pp. ISBN: 978-0-520-25558-6, $19.95

**The Ecological World View**

Charles Krebs

Berkeley:University of California Press, 2008. 592 pp. ISBN: 978-0-520-25479-4, $59.95

**The Use of Nuclear Weapons and the Protection of the Environment during International Armed Conflict**

Erik Koppe

Oxford, UK:Hart Publishing, 2008. 444 pp. ISBN: 978-1-8411-3745-2, $105

**Water Policy in Australia: The Impact of Change and Uncertainty**

Lin Crase

Washington, DC:RFF Press, 2008. 300 pp. ISBN: 978-1-933115-58-0, $70

